# Description of Cardiological Apps From the German App Store: Semiautomated Retrospective App Store Analysis

**DOI:** 10.2196/11753

**Published:** 2018-11-20

**Authors:** Urs-Vito Albrecht, Gerd Hasenfuß, Ute von Jan

**Affiliations:** 1 Peter L Reichertz Institute for Medical Informatics Hannover Medical School Hannover Germany; 2 Department of Cardiology and Pneumology University Medical Center Göttingen Göttingen Germany

**Keywords:** mHealth, mobile health, mobile apps, retrospective app store analysis, cardiology

## Abstract

**Background:**

In the app stores of mobile platforms, consumers are confronted with an enormous number of mobile apps. Over the past few years, considerable research has been undertaken into to identifying, characterizing, and evaluating apps, be it in health-related or other contexts. However, many of these projects are restricted to specific areas of application and offer little flexibility in adapting the applied criteria.

**Objective:**

This paper presents an adaptable method for selecting and characterizing mobile apps listed in a mobile App Store (the Apple App Store). The method is based on filtering processes using predefined criteria, through a semiautomated retrospective App Store analysis (SARASA).

**Methods:**

To illustrate the SARASA process, keyword-based filtering and metadata-based description, review, and ranking steps were applied to a dataset, more specifically, an April 2018 readout of the Medical category of the German App Store, with the aim of obtaining a list of cardiology-related apps.

**Results:**

From the original list of 39,427 apps within the “Medical” category of the App Store on April 14, 2018, 34,382 apps with store descriptions in languages other than German were removed. For the remaining 5045 apps, keywords related to cardiology were applied to filter the output, obtaining a final total of 335 subject-specific apps for further analysis and description.

**Conclusions:**

SARASA provides an easy to use method for applying filtering processes to identify apps matching predefined, formal criteria from app stores. The criteria can be well adapted to the needs of users. Automatic and manual analyses are easily combined when using SARASA. In the future, additional features, such as algorithmic topic analyses, may supplement the process. Although the area of application is currently limited to Apple’s App Store, expansion to other stores is planned. The method stands or falls with the transparency of the app store providers and the manufacturers to make relevant meta-information available. It is up to them to liberalize information and restrict censorship to provide clients, customers, and users truly fair circumstances finding their way around the app market.

## Introduction

### Background

Analyses of software repositories predate modern distribution channels for mobile software [1-3]. In the context of mobile apps, the demand for methods to identify, characterize, and evaluate health-related apps has led to considerable research activity in recent years [4], be it in health-related fields or other areas of application. For many of these projects, there is an emphasis on collecting metadata for apps and evaluating and preparing meaningful information for users [5] to help them select apps that meet their needs. In addition to commercial resources, for example, those that contain (preselected) apps evaluated by experts or user communities, independent scientific analyses (based on peer review) can provide valuable information [6]. In the former case, interested parties must rely on the qualifications and thoroughness of the evaluators, whereas in the latter case, the delay between the point in time when the evaluation was performed and the publication of the results—as it is often customary in science—can lead to incongruence between the information published and reality. Evaluations and ratings from official bodies are only infrequently available because of the sheer number of apps that are listed on the stores [7].

However, there is often only limited information about the methodologies used by those performing the analyses. Scientific approaches try to describe and classify apps based on available information [8-10]. For example, lists of apps may be assigned a ranking [1] or apps may be evaluated in terms of their suitability for specific user groups or specific areas of application [11]. There are also approaches aimed at providing developers with information [12], for example, related to measures they could implement to increase the reach of their apps and thus increase their success [13].

Respective analyses are based on various data sources and data types. For example, metadata about an app can be retrieved directly from the stores, for example, using query interfaces provided by the store operators themselves or Web crawlers. It may not only consist of somewhat unstructured store descriptions but also structured information used for organization and management purposes in the stores. Often, factors such as user ratings [14], update frequency, and other attributes [12] are used. Sometimes data from web-based services or other sources available online, for example, search engine results [9] or Twitter posts [15], are also included in the analyses, depending on the context. The evaluations of the recorded data range from simple metadata evaluations to machine learning–based approaches, which rely on the evaluation or classification of apps, requiring time-consuming, prior training.

### Objectives

This paper presents a method for identifying and describing health apps based on formal criteria. To illustrate the process, we present an evaluation of our proposed semiautomated retrospective App Store analysis (SARASA) [16,17] applied to the German Apple App Store, using manufacturer-provided app descriptions and other metadata, shown in an exemplary manner for apps related to the field of cardiology. Compared with other approaches, SARASA emphasizes the aggregation and filtering of apps, rather than a possible qualitative evaluation. The latter would constitute additional processing based on appropriate methods, exceeding the scope of the work presented here. This study introduces the filtering methodology for apps listed in the App Store and demonstrates an exemplary descriptive evaluation using cardiology-related apps, listed within the “Medical” store category, for which German app descriptions are available. In the final part, the proposed methods are discussed critically.

## Methods

### Principles of the Semiautomated Retrospective App Store Analysis

SARASA describes a multistep procedure, consisting of automated extraction and analysis and manual review and assessment processes, which are described in further detail in the following paragraphs. An example of a viable application of SARASA, as shown in the following sections, is a descriptive evaluation of cardiology-related, German-language apps in the “Medical” category of the German storefront of the App Store.

**Figure 1:**
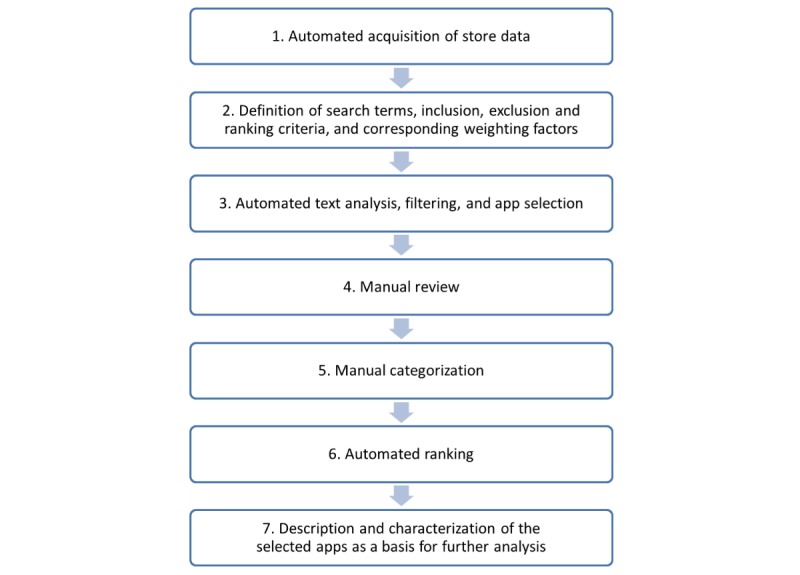
The 7 steps of the semiautomated retrospective app store analysis (SARASA) workflow.

Fundamentally, the SARASA method consists of 7 steps (Figure 1): (1) first, the base data are collected automatically through a total data collection for the desired store categories at a specific time point (step 1); (2) following this, search terms, inclusion and exclusion criteria, and ranking criteria (to which weightings are assigned) are defined, in this example, as mentioned before, for cardiology-related apps (step 2); (3) in the next step, an automated text analysis and filtering is performed to extract a selection of apps from the database. For this purpose, the manufacturer-provided app description texts are used (step 3); (4) the results are then manually validated (step 4) and (5) manually categorized (step 5); (6) following this, the extract is automatically sorted according to the defined ranking criteria (step 6); (7) finally, an app selection with accompanying summary descriptions of the extract is available and may be used for further evaluations as desired, for example, content-based evaluations for assessing app quality (step 7).

### Data Collection

Due to the lack of publicly available, comprehensive, and readily accessible app inventory lists, which also provide full access to metadata, such a list was created for Apple's German App Store on April 14, 2018, using specifically developed, R-based scripts (R Version 3.4.4 [18] with the following libraries: rvest [19], httr [20], jsonlite [21], RSQLite [22], DBI [23], and stringr [24]). The starting points for data acquisition were the German Web pages for the 2 chosen App Store categories: “Medical” and “Health & Fitness.” This allowed for the collection of information about apps provided for health-related purposes. The initial R-based script was used to read the names and unique app IDs of a total of 103,364 apps in the 2 store categories. The associated meta-information (see Table 1) was acquired in the following 24 hours using a second script, which was based on the “iTunes Search Application Interface (API),” provided by the App Store provider. Results were stored in an SQLite-based database to be perused for later evaluations. Varying slightly to this, in the evaluation shown here, only data for apps listed in the “Medical” category were used. In addition, following the initial data collection process, automated language detection processes, based on algorithms published by Google [25], were applied to the store description texts to support filtering by language.

### Definition of Cardiology-Related Search Terms, Inclusion and Exclusion Criteria

SARASA provides a method for identifying apps related to a desired topic based on various criteria that are applied to the available app data. In addition to utilizing the apps’ properties for characterization, for example, based on attributes derived directly from the available metadata, an inclusion or exclusion criterion based on suitable keywords is also possible. To this end, lists of search terms for the desired subject area need to be created before the data can be filtered according to further criteria.

#### Defining Appropriate Keyword Lists

A key element of the selection process is the list of keywords that ultimately influences the app selection for later (manual) fine-tuning. In the sample run of SARASA as presented here, the definition of cardiology-related keywords was based on a list of terms commonly used in cardiology and cardiology-related areas, established through consensus of the authors. This initial list was extended and validated by means of an iterative procedure. For this purpose, functions of the R-package “wordVectors” [26] were used. These make it possible to identify words frequently associated with given terms. The texts of the store descriptions for all 103,364 apps in the “Medical” and “Health & Fitness” categories that were initially selected were processed as follows: Initially, there was a restriction to apps for which a German-language store description could be confirmed using automated algorithms [25]. This analysis was necessary because otherwise, a correct language classification would not have been possible: For a number of apps, the language of the available store descriptions does not match purported language in the corresponding, manufacturer-provided metadata field that denotes the languages in which the app is provided. Filtering the texts by keywords would thus have failed.

The initial list of search terms was extended on the basis of the existing app description texts. For this purpose, the descriptions of apps with recognized German texts that matched the initial list of search terms were preprocessed. Formatting and punctuation marks, digits not enclosed in a word or acronym, and filling words such as articles, number words, and pronouns were removed, and particularly, frequently combined terms were combined to form so-called N-grams (eg, “hoher Blutdruck” = “high blood pressure” and “externer Defibrillator” = external defibrillator). The app descriptions prepared in this manner were then used to fine tune the keyword lists. It is only during this process that we chose to also include app descriptions assigned to the “Health & Fitness” category of the App Store.

**Table 1:** Metadata fields provided by Apple with relevance to our evaluation.

Data field^a^	Description
trackId	Numeric identifier of the app
trackName	App name
features	This is set to “iOSUniversal” if the app will work on all iOS-based devices independent of form factor
supportedDevices	List of possible devices on which the app runs
fileSizeBytes	File size in bytes
artistId	Numeric identifier for the manufacturer
sellerUrl	Link to a website provided by the manufacturer (if available)
price	Price of the app (numeric value without currency specification), example, 1.99
currency	Currency for the *“* price” field
genreIds	Numeric identifiers of the store categories assigned to the app
primaryGenreId	Numeric identifiers for the primary store category
minimumOsVersion	Minimum iOS version required
releaseDate	Date the app was first deployed; corresponds to field “currentVersionReleaseDate” for apps that have not yet been updated
currentVersionReleaseDate	Release date of the currently available, most recent app version
releaseNotes	Additional information provided by the manufacturer, if the app has been updated at least once
description	Full text of the store description
averageUserRating	The average star rating of all versions of an app (if sufficient numbers of ratings are available); may be empty
userRatingCount	The number of ratings of all versions of an app (if there are sufficient ratings); may be empty.

^a^Data fields with differing identifiers but identical content were merged.

This was done to obtain a more comprehensive set of keywords, for example, cardiology-related search terms more commonly used in an amateur context, which would have been more likely to be listed under the store category “Health & Fitness” and could possibly have been overlooked if we had solely restricted ourselves to apps listed in the “Medical” category. For each search term in the initial keyword list, the words most commonly associated with the respective term were recorded. To compile this list, for each occurrence of a keyword, the 10 words or N-grams in its direct proximity (± 5 words) were appended to a list, which was then ordered by the number of occurrences of each word or word combination. For words with an obvious cardiology reference (established through consensus of the authors), again, a list of the 10 most frequently associated terms was compiled. These final word lists were then manually checked by the authors for their potential to extend the keyword list. However, apart from different spellings (eg, words with or without hyphen for compound terms and common typographical errors), there were no significant changes observed for cardiology-related keywords.

For the subsequent automated filtering of all apps with German descriptions, the identified search keywords were then converted into regular expressions (in Perl notation) and optimized (see Textbox 1).

We chose not to use case sensitivity. For example, the partial term “blut[hoc]*druck” thus matched terms “Bluthochdruck” (high blood pressure) and “Blutdruck” (blood pressure) in the filtering process.

#### Definition of Inclusion and Exclusion Criteria

For the final analysis, only apps with German-language store descriptions, for which at least one of the predefined, cardiology-related keywords matched, were retained. Furthermore, for these apps, “Medical” had to be set as either as the primary or secondary category (assigned by the manufacturer). All apps not meeting these criteria were excluded from further analysis.

**Conversion of search keywords into regular expressions in Perl notation. Spaces around the vertical bar characters were only inserted to improve readability and were not part of the actual search string used.**

a[r]{1,2}hythmie[n]* | atrioventrikularklapp|bikuspidalklapp | blut[hoc]*druck | blutgefä[sß]{1,2} | bradykard|cardiol |‌ defibrillat[orin]* |‌ elektrokardiogra[phien]* |‌ erregungsleitungssyst |‌ extrasystol |‌ herzanalys |‌ herzanf[aä]+ll |‌ herzbeschwerd |‌ herz[druck]*massag |‌ herz[er]*krank |‌ herzfehler |‌ herzfit |‌ herzfrequenz |‌ herzfunktion |‌ herzgesund |‌ herzgeweb |‌ herzinfarkt |‌ herzinsuffizien |‌ herzkamm |‌ herzkatheter |‌ herzklapp |‌ herz[kranz]*gefä[sß]* |‌ herz[-]*kreislauf[-]* |‌ herz[minute-]*volum |‌ herz[-]*monitor |‌ herz[-]*patient |‌ herzprobl |‌ herzras |‌ herzrhythmus |‌ herzschl[aä]+g |‌ herzschrittmach |‌ herzschw[aä]+ch |‌ herzspezialist |‌ herzstiftung |‌ herzstillstand |‌ herztagebuch |‌ herztest |‌ herztod |‌ herztransplantation |‌ herzzyklus |‌ hypertens |‌ hyperton |‌ kardial |‌ kardiol |‌ klappenprolaps |‌ koronar |‌ kreislaufforsch |‌ kreislaufstillstand |‌ kreislaufsystem |‌ mitralklapp |‌ myo[ck]+ard |‌‌ pulmonalarterie |‌‌ pulmonalklapp |‌ schlagader |‌ systol |‌ trikuspidalklapp |‌ ventrikel |‌ vorhof | diastol |‌ bekg[s]* |‌ bgefä[sß]{1,2}w* |‌ bw*aort[aen]+w*

### Added Information: Readability Indices

For each app remaining in the analysis process, the readability index according to Flesch, that is, the Flesch Readability Ease ([27,28], with adaptations by Amstad for German language texts [29]), was determined using the functions provided in R (Version 3.4.4, [18]) via the koRpus package [30]. The aim was to offer an additional descriptive feature related to text difficulty for assessing the suitability and comprehensiveness of the store descriptions for specific target groups. In preparation, the descriptions were edited with regard to possible misinterpretations of sentence lengths (number of words), especially in the context of bullet lists; for example, we used regular expression-based search and replace operations matching commonly used bullet point characters to identify and replace these with periods if the previous bullet point had not been ended by appropriate punctuation. Similar to approaches described elsewhere [31], this preparation was done to avoid a grossly incorrect calculation of the readability index, which includes, among other things, recognizing sentence lengths. As the readability index developed by Flesch [27,28] has—in addition to the above-mentioned adaptation to the German language—been adapted to various other languages, we chose it over several other candidates, for example, for German-language texts, the “Wiener Sachtextformeln” by Bamberger and Vanecek [32] or the “Läsbarhetsindex” LIX, which was originally developed for Swedish-language texts [33] but is also applicable to those in German language. Furthermore, despite literature about the score’s validity being somewhat limited, it is nevertheless widely employed (sometimes even as a legal requirement, eg, with insurance contracts being required to have a Flesch readability index of at least 45 in Florida state law [34]). Although not being fully comparable across languages, the diverse availability of the Flesch index provides a possible avenue to adapt the implemented methods to analyses of apps in languages other than German: This would only require adjusting 1 parameter in the analysis pipeline, without demanding additional changes to the code base.

### Manual Review of the Cardiology-Related Set of Apps

The cardiology-related apps, as determined in the previous steps, were manually validated by the authors, with any uncertainties being resolved by discussion. As the aim was to identify apps relevant to the field of cardiology, all apps remotely addressing cardiological issues were included. As we did not want to limit ourselves to a specific target audience, apps deemed acceptable included those for cardiologists or other medical specialties as well as apps for patients or health conscious users, the latter also including apps one might use in a preventive or rehabilitative context. With this in mind, with the exception of 1 app, in which the search term “Vorhof” (atrium) was not employed in a cardiology-related context but as a part of a term related to other anatomical structures (“Kehlkopfvorhof”, literal translation: atrium of the larynx), there were no obvious mismatches to terms not used in cardiology-related contexts. Some apps related to cardiology but intended for use in a veterinary environment could have been excluded, and there were also apps trying to influence their users’ heart rate, for example, by means of meditation or other apps with a rather alternative approach to the subject. For cardiologists applying SARASA to identify apps for their specific professional needs, these apps would of course not be acceptable and would be eliminated. However, in our evaluation, these apps were not removed to create a realistic application scenario that can reproduce a manual keyword-based search within the app store, and we chose instead to differentiate them via the manual categorization process described in the following sections.

### Manual Categorization and (Metadata-Based) Evaluation

The remaining apps were then classified manually by the authors, according to function types and subject areas.

#### App Categorization by Function Type

The 22 function types developed in the CHARISMHA (Chances and Risks of Mobile Health Apps) study [35], which can be grouped into 6 superordinate categories (Table 2), were used to subdivide the apps. These function types are to be seen independently of the “cardiology” application case considered here and should generally be applicable to apps that are used in health contexts.

#### Classification of the Apps by Topics

The groups of topics used for classifying the apps with respect to their subject areas were developed in a discussion between the authors. In addition to the function types mentioned in the previous step, which allow for a subdivision independent of the app’s application area, a classification method focusing on the respective subject area and its facets, in this case, cardiology, is of advantage. For the example shown here, the apps were manually assigned to a set of defined topic groups (Table 3). Disputed topic assignments were clarified by discussion between the authors. Especially in cases with potentially overlapping topics, emphasis was placed on reaching a consensus about the main topic of each app.

### Ranking the Apps by Predefined Criteria

In addition to the aforementioned filtering and review procedures, SARASA also provides a ranking mechanism with the intent to support presorting for manual processing. This mechanism presents apps that best match a manually determined and adaptable set of criteria in an order that displays apps that conform better to certain characteristics more prominently. To this end, predefined (and weighted) ranking criteria are used, relying on attributes either directly deducible from the metadata or calculated by various means.

**Table 2:** Function-related types as defined in the CHARISMHA study, including their superordinate categories, which were used in our manual classification [35].

Category		Description
**Category: provision of information**		
	News	News apps, for example, for professional newspapers or news portals or for patient organizations
	Reference	Apps that provide users with knowledge on health-related topics (eg, reference material)
	Learning material	Apps that provide learning and teaching materials for education and training
	Player/viewer	An app that permits playing or viewing media (eg, music, image data, and videos)
	Broker	Apps that provide targeted information based on collected data (eg, location-based services)
**Category: data acquisition, processing, and evaluation**		
	Decision support	Apps that support decision making based on collected data, based on the definition of decision support according to the study by Shortliffe and Cimino [36]
	Calculator	Apps that perform calculations
	Meter	Apps for immediate measurement of phenomena and characteristics not immediately accessible otherwise, for example, pulse measurement via the camera of the mobile device
	Monitor	An app that may either serve as a measuring tool of its own or connects to a measuring device that is designed for multiple measurements of vital functions and stores them in a diary
	Surveillance/tracker	Apps that automatically and continuously capture certain parameters in the background but do not interpret the data in a medical sense
**Category: administrative use**		
	Administration	Apps for managing administrative data
**Category: calendar and appointment-related apps**		
	Diary	Apps used for detailed data collection and tracking
	Reminder	Apps that remind you of specific tasks
	Calendar	Apps that are used to display and manage health-related events, for example, appointments, in the form of daily, weekly, or monthly overviews
**Category: support**		
	Utility/aid	Apps that can be used as aids and help users to compensate for existing personal limitations (eg, hearing or vision problems)
	Coach	Apps that teach users an activity and help them to carry it out
	Health manager	Apps that are designed to continuously support users in health matters. A combination of several function types is required for an app to be assigned to this function type
**Category: other**		
	Actuator	Apps that produce a direct physical impact in the form of mechanical motion or other physical effects
	Communicator	Apps that are used for communication and getting into contact with others
	Game	Apps that are used for pleasure, relaxation, and enjoyment
	Store	Apps that offer opportunities to buy or sell goods and services
	Other	All apps that cannot be assigned to any of the aforementioned function types

The selection of ranking criteria used in the example evaluation (Table 4) was chosen with the intention, among other things, of taking into account both transparency on the part of the manufacturer as well as (available) user evaluations. The ranking criteria and their assigned weighting factors are, however, freely adaptable, depending on the chosen topic and the objective of the evaluation being performed. As presented here, the inclusion of references to a possible medical device (or the explicit exclusion of this) in the store description, which we also used as a ranking criterion, may at least reflect a basic understanding of the associated problems on the manufacturer's part. Similar to a comprehensive store description and the provision of adequate information about the manufacturers themselves (eg, availability of an associated website for the app or the manufacturer), this examines the transparency of information provided to users. If available, user ratings are also included. It should be noted, however, that user ratings were only available for relatively few apps in the store, and even for these, there were only a limited number of apps that had obtained a significant number of ratings; this may be the reason to assign lesser weight to these factors in the future.

**Table 3:** Definition of the app-related topics, specifically for a cardiology context.

Topic	Description
Atlases	Dedicated teaching, learning, and reference works, for example, anatomy atlases
Blood pressure	Apps containing content and functions that can help with blood pressure management, for example
Conferences	Apps related to organizing one’s conference visit or obtaining information about a conference
ECG^a^	Contents and functions relating to ECG
Nutrition	Nutritional content, for example, on diets (including nutrients) and nutrition-dependent health aspects or disorders
Fitness	Apps that promote fitness content and functions
Women	Apps specifically targeting women
Health data	Apps for the recording, monitoring, and analyzing of health-related data (eg, vital signs)
Communication	Apps with a communicative character, for example, for the exchange of information between medical staff and patients, within patient groups, and online communities
Medication	Medication-related apps
Complementary medicine	Apps that adopt an alternative medical approach (eg, acupuncture, acupressure, meditation, complementary medicine)
Neurology	Apps containing neurology-related content and functionalities
Emergencies	Apps for emergency medical or first aid use
Medical practice or hospital	Apps to be used in medical practice or hospital settings
Psyche	Apps covering psychological and psychiatric issues
Sleep	Apps to be used in sleep-related contexts
Metabolism	Apps specifically designed for use in managing metabolic disorders (eg, diabetes or other metabolic diseases)
Animals	Apps that have a cardiology reference but are intended for use in the field of veterinary medicine

^a^ECG: electrocardiography.

**Table 4:** Ranking criteria with weighting factor (percentage of the overall score), item name, description and condition to be fulfilled, or their explanation.

Variable	Source	Description	Score (%)
medicalDevice	Keyword-based evaluation of the store description (0: no keywords mentioned, 1: entry made)	The regulatory status (medical device) of the app is mentioned or explicitly excluded in the store description or there is mention of a seal of approval (reference to CE^a^, FDA^b^, medical device, or a seal of approval)	20
descriptionLength	Calculated value (number of characters)	Length of the store description	20
averageUserRating	Store metadata (from (0, maximum scoring reached) normalized to value range [0,1])	Average rating of all versions of the app	15
userRatingCount	Store metadata (from (0, maximum number of ratings) normalized to value range [0,1])	Overall number of user ratings that were obtained (for all versions)	15
sellerUrl	Evaluation of the metadata field sellerUrl for a valid URL (defined as nonempty and unequal to *http://* or *https://* without any further information); 0: invalid or empty, 1: valid URL	Link to a website (eg, a manufacturer’s homepage or a Web page for the app) has been provided	10
releaseNotesLength	Calculated value (between (0, maximum number of characters) normalized to [0,1])	Length of the release notes, if available (prerequisite: at least one update, as only then must the field be set)	10
actuality	Calculated value including the time span between publication and readout time (from (0, maximum time span) normalized to 1-[0,1])	Whether the app is up to date	10

^a^CE: Conformité Européenne.

^b^FDA: Food and Drug Administration.

**Figure 2:**
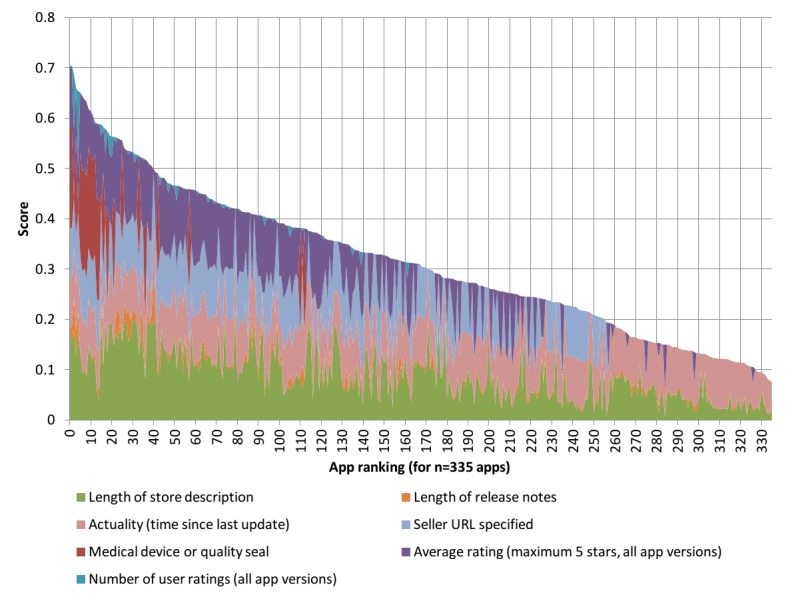
Actual contribution of the currently used ranking factors to the ranking of 335 cardiology-related apps.

Ranking the apps, in this case, based on a score calculated using the attributes and weighting factors defined in Table 4, is intended to support those interested in making an app selection with the help of SARASA. This is particularly useful in the case of a large number of possible results by first drawing attention to apps that are deemed to be particularly relevant depending on conformity to the chosen characteristics. An app presenting with an ideal rating for all attributes contributing to the score would—theoretically—achieve a score of 1.0. In reality, however, the maximum score achieved will usually be much less (eg, 0.7 for our cardiology-related sample evaluation in Figure 2). However, the calculated score and the factors contributing to it are not meant to be used for automatically evaluating app quality- or content-related aspects of the apps.

## Results

### Automated Filtering and App Selection

**Figure 3:**
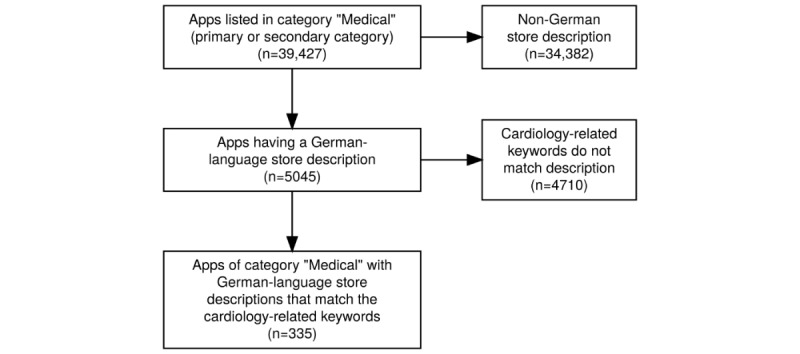
Acquisition and keyword-based selection process for the 335 cardiology-related apps.

As basis for the descriptive statistics presented here, German-language, cardiology-related apps were selected using the aforementioned processes. Originally, there were 39,427 apps listed within the “Medical” category on April 14, 2018. First, 34,382 apps with store descriptions in languages other than German were removed, and for the remaining 5045 apps, the selected keywords were used to further filter the output, obtaining a final total of 335 apps, related to cardiology, for further analysis (see Figure 3).

### Cardiology-Related Apps: Descriptive Statistics

#### General App Demographics

For an initial overview and comparison, descriptive statistics were first calculated for all 39,427 apps of the “Medical” category as well as for the 5045 apps with German-language store descriptions and the 335 apps with matches for the cardiology keywords (Table 5).

German-language apps, as well as those related to cardiology, were on the market slightly longer on average at 32.58 (interquartile range [IQR] 33.35) and 39.25 months (IQR 48.39), respectively, than all apps in the “Medical” category (median 28.22, IQR 34.89).

German-language apps in the “Medical” category (median 11.07 months, IQR 22.51) or with a cardiology reference (median 7.73, IQR 20.20) were updated more than usual, compared with the “Medical” store category (median 12.98 months, IQR 22.32).

**Table 5:** App demography in comparison: apps within the “Medical” category versus those for which a German-language store description was provided versus those having a cardiology reference.

App demographics		All apps assigned to the “Medical” category (N=39,427)	Apps assigned to the “Medical” category that also have a German-language store description (N=5045)	Apps selected via the cardiology keywords (N=335)
Overall age of the apps in months, median (IQR^a^)		28.22 (34.89)	32.58 (33.35)	39.25 (48.39)
Age in months (current version only), median (IQR)		12.98 (22.32)	11.07 (22.51)	7.73 (20.20)
File size in megabytes, median (IQR)		22.56 (36.08)	24.65 (30.37)	30.25 (53.58)
**Price in Euros (€)**				
	Number of paid apps and percentage of total, n (%)	6838 (17.34)	846 (16.77)	91 (27.2)
	Price, median (IQR)	3.99 (7.70)	3.49 (3.20)	3.49 (4.70)
	Price range (€)	0.49-1099.99	0.49-499.99	0.49-249.99
Length of the store description (number of characters), median (IQR)		757 (1048.50)	921 (1502.00)	1630 (1585.50)
**Star ratings (current version)**				
	Rated apps, n (%)	2072 (5.26)	1408 (27.91)	144 (43.0)
	Median rating (IQR)	4.50 (2.00)	4.50 (2.00)	4.50 (2.00)
	Maximum number of ratings (n)	6900	6900	645
	Number of ratings, median (IQR)	2.00 (3.00)	2.00 (5.00)	3.00 (8.00)
**Overall star ratings (all versions)**				
	Rated apps, n (%)	2581 (6.55)	1681 (33.32)	173 (51.6)
	Median rating (IQR)	4.00 (2.00)	4.00 (2.00)	4.00 (1.50)
	Maximum number of ratings (n)	22,153	22,153	6881
	Number of ratings, median (IQR)	6.00 (18.00)	7.00 (22.00)	14.00 (49.00)

^a^IQR: interquartile range.

With regard to the file sizes, cardiological apps seem to be somewhat larger in median (median 30.25 megabytes, IQR 53.58) than those without restriction of the field of application (all apps in category “Medical”: median 22.56 megabytes, IQR 36.08; German-language store description: median 24.65 megabytes, IQR 30.37). It is conceivable that this is influenced by the contents included for apps in the field of cardiology. For example, a higher proportion of reference works with somewhat larger amounts of texts and multimedia content may exert an influence here.

With regard to app pricing, a higher proportion of paid apps (27.2%, 91/335) is seen in those related to cardiology than in other medical apps (all medical apps: 17.34%, 6838/39,427; apps with German description: 17.13%, 864/5045). Although only about every sixth app in the medical category requires a purchase, this is necessary for slightly more than every fourth app in cardiology-related apps with a German store description.

It is also noteworthy that apps in the “Medical” category (with a median of 757 characters, IQR 1048.5) have shorter description texts overall than those in German (median 921 characters, IQR 1502) and, in particular, cardiology-related apps (median 1630, IQR 1585.5). Although German-language texts are generally known for being longer compared with, for example, English-language texts, this does not explain the much greater length of descriptions of cardiology-related apps; it is also in this instance that specific cardiology-related peculiarities are speculated as a potential cause.

With regard to the app ratings given by users for the most recent versions available through the store, there were only a small number of apps that had received any ratings at all. That being said, there was a much larger proportion of current ratings for German-language apps (27.91%, 1408/5045) and apps found using the cardiology-related keywords (43,0%, 144/335) compared with all apps (5.26%, 2072/39,427) listed in the “Medical” category. Looking at the ratings given for all versions of the apps, the percentage of apps rated for all medical apps was 6.55% (2581/39,427) compared with 33.32% (1681/5045) for German-language apps and 51.6% (173/335) for those relating to cardiology. Median ratings differ only marginally.

#### Manufacturers

The vast majority of the vendors of the 335 selected apps are represented with only a single app in the app selection (Table 6). This can be determined based on the manufacturers’ names and identification numbers. Some vendors provide more than 1 app with cardiology-related content and German-language store description in the store. Especially for manufacturers who offer several apps within this field, there are often only small variations in the content of the apps provided. These can be separately listed as lite or full versions of apps or versions for different form factors (iPhone and iPad) that are also shown separately. Also noticeable in this context are manufacturers who provide several apps covering cardiology conferences or various reference books or atlases on cardiology-related topics.

#### Hardware and Software Requirements Specified by the Manufacturers

The extent to which an app can be used depends, among other things, on the technical requirements it demands from the devices on which it should be run. Devices with iOS 9 or 10 are still represented in relevant figures. According to Apple, the (at the time of this writing) current iOS 11 version was installed on 81% of all devices at the end of May 2018. This does, however, mean that approximately one-fifth of the devices in use were not yet equipped for apps requiring this version. Apps that only require iOS 6 or older versions can hardly be expected to have been updated (see Table 7; the “Cumulative percentage” column indicates the percentage of apps listed under category “Medical” that can be used with a device running the corresponding iOS version).

As to usability on different form factors, 175 of the 335 apps stated that they could be used universally, that is, on all device types. The remaining 160 apps, on the other hand, require specific device types.

#### Automated Text Complexity Analysis of Store Description Texts

**Figure 4:**
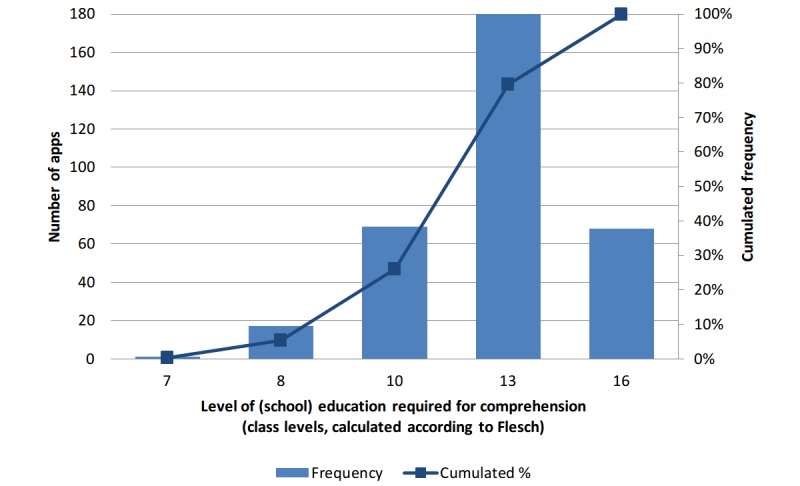
Distribution of the educational levels required for comprehending the description texts of apps related to cardiology.

For about one-quarter of the apps (26.0%, 87/335), based on the automatically derived readability scores, only relatively low educational standards were required for potential users: On the basis of the available description texts for the corresponding apps, a maximum of 10 school years was required to comprehend the texts. However, just over half of the apps (53.7%, 180/335) required a high school diploma level, and for about one-fifth of the apps (20.3%, 68/335), the results of the text complexity analysis according to Flesch [27,28] (with adaptations for German-language texts according to Amstad [29]) indicated a level of difficulty going beyond that (Figure 4). It should be noted that the text complexity analysis as described here does not allow any statement as to whether or not the texts were actually grammatically correct. During a manual check, a few apps were identified whose description texts had obviously been automatically translated from other languages.

**Table 6:** The number of apps per manufacturer.

Apps provided by a single manufacturer (n)	Manufacturers with n apps in the store (n)	Percentage of 335 apps
1	178	53.1
2	43	25.7
3	4	3.6
4	3	3.6
6	2	3.6
8	1	2.4
9	3	8.1

**Table 7:** Description of the 335 cardiology-related apps stratified by their minimally required iOS versions.

iOS version^a^	First date of release [37]	End of life for the following devices [37]	Apps (n)	Proportion (%)	Age (in days) on the readout date		Cumulative percentage (%)
					Minimum age	Median (IQR^a^)	
3.x	June 17, 2009	iOS 3.1.3: iPhone 1st generation, iPod touch 1	2	0.6	2744.9	2892.11 (147.17)	0.6
4.x	June 21, 2010	iOS 4.2.1: iPhone 3G, iPod touch 2	6	1.8	1575.9	2076.26 (216.79)	2.4
5.x	October 12, 2011	iOS 5.1.1: iPad 1st generation, iPod touch 3	11	3.3	113.6	1599.42 (474.23)	5.7
6.x	September 19, 2012	iOS 6.1.6: iPhone 3GS, iPod touch 4	31	9.3	222.4	998.73 (495.18)	14.9
7.x	September 18, 2013	iOS 7.1.2: iPhone 4	47	14.0	5.7	586.39 (627.97)	29,0
8.x	September 17, 2014	N/A^b^	119	35.5	0.9	276.84 (329.45)	64.5
9.x	September 16, 2015	iOS 9.3.5: iPad 2 and 3, iPad Mini 1, iPhone 4S, iPod touch 5	79	23.6	0.1	68.25 (148.08)	88.1
10.x	September 13, 2016	iOS 10.3.3: iPad 4, iPhone 5 and 5C	33	9.9	2.8	50.84 (130.24)	97.9
11.x	September 19, 2017	N/A	7	2.1	33.2	83.69 (89.56)	100

^a^Minimum version. For clarity, the information is summarized according to the main iOS versions.

^b^N/A: not applicable.

### Comparisons of Function Type Groups and Subject Areas as Well as Cardiology-Related Keyword Groups

With regard to function types (as defined in Table 2), the cardiology-related apps were predominately apps that provide information (n=130), support users (n=72), or collect, provide, or evaluate data (n=60, Multimedia Appendix 1). Apps that are designed to provide information often do this by instructing users on how to behave or take action in emergencies (n=32). Apps for medical conferences (n=14), medical reference works (eg, anatomy atlases, n=10) and other apps that cannot be assigned to a specific topic are also frequently found in the context of information provision. In the cardiology-related app group, health data are primarily collected and processed by apps falling into the functional categories of *support* (n=22) and *data acquisition, processing, and evaluation* (n=28). Support apps offer functions that go beyond the mere recording and processing of data and strive to achieve an added value for the user. Of these, apps belonging to the field of complementary medicine (n=24) are noteworthy. For example, although not exactly targeting cardiology in a strict sense, these often try to influence parameters such as heart rate or blood pressure with acupressure or mediation and were therefore also included on the basis of hits when employing the corresponding keywords.

Among other things, apps classified under *calendar and appointment related* often offer functions that remind users to record their data or help with adherence to prescribed medication. These can include blood pressure and diabetes diaries that record cardiology-relevant parameters or remind users of other measurements or medications.

### Categorization of Apps by Function Type, Topics, and Associated Metadata

When looking at app demography stratified by manually assigned function types, some particularities are noticeable for apps related to cardiology (Multimedia Appendix 2). Based on the median number of months since the original publication of the apps, apps that deal with data acquisition, provision, and evaluation (44.59, IQR 41.07) or provide calendar- and time-related functions (53.38, IQR 36.63) have been on the market the longest. With median updates dating back 7.73 months (IQR: 20.20), updates to the analyzed cardiology-related apps are significantly more recent than for other German-language apps in the “Medical” category (12.98, IQR 22.32, Table 5). Updates to apps that serve to provide information are slightly less recent with a median of 9.47 months (IQR 20.30), despite the fact that, for these apps, it would be especially desirable to regularly check that the content is up to date.

As expected, the size (in megabytes) of apps that provide information (median 28.79, IQR 54.58) or help guide users to do exercises, for example coaching apps (function type *support*, median 52.59, IQR 51.28), and thus include additional text and multimedia content, exceeds the size of apps that only evaluate and process recorded data (16.66, IQR 36.27) or offer management functions (9.28, IQR 53.79).

The available ratings of the cardiology-related apps are unremarkable. This is regardless of their assigned function type and whether only the current or all versions provided in the store are considered. Notable is the significantly higher proportion of apps rated (between 33.8% of the current versions of apps providing information and 78% of all versions of apps of the “Support” function type), when compared with the values otherwise usual in the medical category (proportion of apps with ratings for the current version: 5.26%, all versions: 6.55%); the median ratings differ only marginally in comparison.

Similarly, differences between the groups with respect to the distribution of prices within the groups are unremarkable. With the exception of a larger proportion of paid cardiology-related apps (27.2%, 91/335)—compared with all apps in the medical category (17.34% paid apps, 6838/39,427) and those with German-language descriptions (17.13% paid apps, 864/5045)—there are only minor differences in median prices, which can mainly be explained by some particularly expensive “outliers.”

In terms of the 19 manually assigned subject areas, apps that use health data in the broadest sense are the most common (20.0%, 67/335). Apps for use in emergencies (13.1%, 44/335) and those where blood pressure (10.7%, 36/335) or complementary medicine (9.3%, 31/335,) are also seen frequently. Overall, 13.4% (45/335) of the apps could not be assigned to a specific topic (Multimedia Appendix 3).

Observing the median, the sample apps originally appeared 4 to 5 years ago, and apps belonging to the field of complementary medicine had been published approximately 6 to 8 month (58.49, IQR 47.48) before the blood pressure apps (52.68, IQR 37.49), metabolic apps (52.11, IQR 48.71), and ECG apps (50.74, IQR 31.85) contained in the sample.

Apps are updated relatively frequently (median age of the currently available version in month 7.73, IQR 20.20). Exceptions are apps for organizing one’s conference participation or apps that provide information about such events after the conference has taken place.

Apps from the ECG domain exhibit noticeable differences in terms of pricing. Almost every second app (47%, 7/15) is subject to a fee. With a median price of €20.99 (IQR 119.85) and a maximum price of €249.99, these apps are also significantly more expensive when compared with the other thematic areas.

With respect to the length of provided store descriptions, apps aimed at laymen and patients, in particular, for example, for complementary medicine (2600, IQR 736.50), metabolism-related apps (2003.50, IQR 1268.00), or those to be used in conjunction with medication (2023.00, IQR 1829.50), tend to have more extensive descriptions (represented by the number of characters) than other cardiology apps (1630, IQR 1585.50).

Apps for cardiological issues were rated more frequently than other apps in the “Medical” category (6.55%, 2581/39,427, Table 5) and apps with German descriptions (33.32%, 1681/5045, Table 5). More than every second (51.6%, 173/335) app has a star rating. In some areas (blood pressure and metabolism), as many as 3 of 4 apps were rated by users.

## Discussion

### Principal Findings

The aim of this study was to present a low-threshold solution for store analysis, which provides flexible support in the selection of apps, despite changing requirement profiles (previous knowledge of the interested parties, variability of use cases, or application scenarios) and without additional effort. The manual assignment of function types and topics for descriptive purposes is to be understood as an optional step until it can be carried out automatically. The analysis not only takes into account all available metadata, especially app description texts, but also other attributes such as average user ratings. Although others such as Berardi et al [8], for example, strive for app classification using a somewhat similar approach, SARASA is currently designed as a tool to prepare for further manual evaluations. SARASA enables filtering of larger sets of apps, for example, entire store categories, based on the entirety of available data. To this end, it also evaluates available store descriptions and creates a ranking of the apps based on selected attributes (multiplied by weighting factors), making it possible to sort apps according to subjective, analyst-defined relevance. Depending on the problem, both the attributes to be evaluated and the weighting factors can be adjusted as required when using SARASA. Additions, such as the inclusion of an automated text complexity analysis for app descriptions in the ranking, for example, to create a focus on specific target groups, are easily possible. In particular, with regard to urgent questions of quality and security and regarding the suitability [7,38-40] and benefits [41] of apps, additions such as source code analyses or the recording of API calls [42] and requested authorizations [43] are to be integrated in future.

At the current stage of development, the approach presented is subject to several limitations, the knowledge of which is essential for assessing the method. These are explained in further details below. Much of future work on SARASA will have to take these aspects into account. In the course of this, additional modules may be included into SARASA.

### Limitations

#### Platforms, Manufacturers, and Their Commitment to Transparency

The incorporation of various characteristics into analyses on a larger scale is strongly dependent on the willingness of the respective bodies to be transparent. For example, analyzing app installation archives may require the ability to download and analyze installation archives, without actually installing them on a physical device, but this may not be possible for all mobile platforms. In addition, not all app stores provide the same amount of access to desired attributes, for example, regarding required app permissions and numbers of downloads, the willingness of store providers to be transparent is also somewhat limited. For this reason, the analysis according to the SARASA scheme is currently limited to a single App Store (Apple). In our example evaluation, the German-language storefront was used to obtain the data. Evaluating other regional storefronts might have led to larger numbers of apps for which a more time-consuming manual evaluation would have been necessary. Using the ranking methodology, possibly with further adaptations to the attributes and ranking factors used, may be essential to still keep filtered results manageable, for example, by only evaluating a specific proportion of the top-ranked apps (based on the calculated score).

For stores of other platforms, for example the Play Store provided by Google for Android, an adaptation of the readout and—to a lesser extent—the evaluation routines will be necessary. For example, there is no official interface available for Google's Play Store that would allow full capture of the store or individual categories. In addition, some of the attributes provided in the stores differ between platforms, making it difficult to compare results of the SARASA method when applied to apps on different platforms.

#### Sampling Bias

The method presented in this study, a complete survey of the desired store categories, demonstrates a substantial reduction in, often criticized, sampling bias [44] in the evaluation of app-related data. It is conceivable, however, that manufacturers inappropriately assign other categories to their apps (eg, combinations of the store categories “Reference,” “Book,” or “Lifestyle” as primary and secondary categories for a reference work aimed at laymen)—because of uncertainties—and thus, some apps may be wrongly excluded from a SARASA-based analysis. Despite this, such cases would still yield a matching result based on keywords, if applied to data from other categories.

Regardless, the SARASA method is prone to underrepresentation of certain apps if the keywords chosen for selecting the apps do not adequately cover the desired subject. It could be argued that the proposed approach may not guarantee the identification of all suitable apps: For example, although nutritional and many other types of apps may also exert influence on cardiological parameters and thus be relevant in cardiology-related use cases, these will not be returned if their descriptions do not match any of the chosen search terms; adding corresponding terms to our search would, however, have been outside the scope of our presented work, as our aim was not to even identify apps for which the manufacturers had failed to specify a corresponding connection or purpose. In this instance, the limitations are comparable with those of a systematic literature search in review articles. Here, a strategy is used, searching for potentially relevant literature in databases; following the initial search, the results of course need to be evaluated manually. These reviews, however, do not commonly aim at determining whether or not there were potential matches that were missed, and which exactly were these; measures such as sensitivity and specificity, which are indispensable in diagnostic studies, are not common in literature searches, and this also holds true for the SARASA method. We believe that the comprehensiveness of the results for both literature and app searches, as they were described in this paper, can be derived from the comprehensive and easily verifiable search strategy, which includes a transparent specification of the search keywords, inclusion and exclusion criteria, and so on.

Selecting apps based on our methodology may also be favorable when compared with solely searching for apps based on search APIs or Web interfaces, as they are services provided by the respective app stores. In Apple’s case, for example, there is currently a maximum of 200 search results (in this case, apps) for keyword-based searches when using the provided search API [45], and it is somewhat tedious to perform searches for multiple keywords; our approach, at least for iOS-based apps, does not suffer from such restrictions.

Still, a bias may be introduced when applying the methodology to data acquired from other App Stores in the future. Much will depend on whether readout routines for these stores allow access to all apps in the desired store categories, rather than restricting access to so-called top apps. A complete survey of an entire app store’s content, which could, for example, counteract the aforementioned bias of incorrect category allocation on the part of the manufacturers, will not be expedient simply because of the scope. Here, the platform providers’ desire for transparency also plays a decisive role.

#### Language-Dependent Aspects

When selecting storefronts provided for other (language) regions (eg, this is possible when reading out the apps via the API provided by Apple), a significant variation of the results is to be expected. For example, if English-language apps are included in the evaluation, the number of apps selected increases many times over. As the purpose of the study was purely to illustrate the filtering and classification of apps using SARASA, we decided to confine ourselves to the described restrictions in database acquisition. In the future, the processes for other app stores or linguistic and geographical regions will have to be adapted so that universal statements beyond geographical or language borders can be made. In addition, changing the language or adding additional languages to SARASA-based evaluations will require adjustments that go beyond simply translating the search terms. For example, although the German language is known for the use of, often rather lengthy, compound terms, N-grams will be more relevant in other languages. Aspects such as these must then be taken into account within the search.

#### Machine Learning

At present, the assignment to define function types and subject areas was done manually to classify prefiltered apps based on language and keywords. For topics or inquiries that lead to a number of hits that significantly exceed the number demonstrated here, an automation of these assignments would be desirable. In an initial attempt to achieve this by means of keyword-based assignment, only little correlation with the manually defined assignments was observed. This is why the strategy was not pursued further in the context of the work presented here.

In spite of advantages, a more efficient procedure, for example, via a machine learning–based assignment, would have initially increased the work required (eg, due to the need for manually preclassifying training data). Nevertheless, it is planned to implement natural language processing (NLP)-based methods (specifically, topic analysis) in the future to enable at least a basic assignment. This idea seems particularly promising for the manual definition and assignment of subject areas. These would otherwise have to be redefined and discussed when using SARASA if the selected area of application changes. A topic analysis that would be an automatic definition of certain thematic subareas from the initially filtered apps and the ability to reliably assign the apps to these subareas would be helpful and should therefore be a goal of future developments of SARASA. The extent to which a successful assignment to the function types, known from the study by Albrecht et al [35], by means of such an approach is possible or whether this will indeed remain a meaningful part of SARASA-based analyses in the future must also be part of future investigations. A preliminary topic analysis [46,47] carried out for testing purposes, based on the available store descriptions, indicated that at least a mixed assignment of topics and function types is possible; this may already be sufficient.

#### Ranking

The ranking is strongly dependent on the analyst-defined values and factors used. Depending on the desired filtering objective or target group, it may make sense to adjust the ranking factors and/or to include the score calculated for the ranking as an additional filter criterion in the SARASA process. This could exclude apps that fall below a minimum score, defined from the outset. For example, Berardi et al [8] also use attributes such as the size of an app, for which they assume significance in relation to the complexity of an app or the scope of its content. Depending on the objective—should the analysis apply, for example, to learning apps or reference works—larger apps could then be ranked more prominently.

#### Text Complexity Analysis

The automated text complexity analysis, outlined only briefly in this paper, is subject to certain limitations as well, especially for nonstandard texts. Only in a few cases do the authors of the description texts follow the conventions commonly used for continuous texts in scientific or journalistic fields. Algorithms for determining text complexity, however, are usually specially standardized for such texts, expecting, among other things, a certain minimum length. Particularly with regard to punctuation and formatting, but also addressing texts that are (too) short in length, peculiarities or deviations are to be noted in app descriptions, which can have a negative influence on the automated analysis. Our analysis tried to eliminate the most common problems relating to punctuation and so on. Nevertheless, it cannot be ruled out that, for example, missing sentence points or formatting characters recognized as punctuation elements may cause the results to be skewed because the (recognized) sentence length (eg, represented by the number of words in a sentence) plays an essential role in the calculation of many readability formulas. It is, however, not possible to counteract the widespread problem of texts being too short. In the future, it may therefore make sense to additionally resort to other measures such as lexical diversity (calculated based on the number of different words/terms in a text) in addition to a pure text complexity analysis, if corresponding statements on text complexity or comprehensibility that can be derived from the texts are to remain part of SARASA.

#### Usability Aspects of the Semiautomated Retrospective App Store Analysis

For the future, it is planned to evolve the filtering process. At present, filtering can only be adjusted by parameterization in the R-based scripts. The aim would be to create a shiny frontend [48], that is, a user interface that can be operated via Web browser, even without programming knowledge, thus making the filtering process available to a wider audience.

### Conclusions

SARASA is a method for filtering app store data according to formal criteria and accompanying description of the extract using common statistical measures. The filter results contain a selection of apps that can be passed through subsequent processing steps, which can, for example, following manual review of the list, consist of content-based quality assessments. SARASA allows the implementation of a flexible filter strategy, adaptable to the needs of the user. Automatic and manual analyses are easily combined when using SARASA. In the future, current functions will be supplemented by additional features, such as algorithmic topic analyses or sentiment analyses of user-provided comments (whenever user ratings are to be included as part of the analysis pipeline). The area of application is currently only limited to Apple's App Store, although expansion to other stores is planned. The method stands or falls with the transparency of app store providers and manufacturers, and their will to make relevant meta-information available. It is up to them to liberalize information and restrict censorship to provide clients, customers, and users truly fair circumstances finding their way around the app market. However, based on the available information, a fully automated selection, assessment, and recommendation of apps is not the aim of the SARASA method: The final decision about whether an app really has desired characteristics can only be made by reviewing and analyzing the metadata provided on the store as well as the apps themselves, which, for the time being, is not feasible without human intervention.

## Appendix

Multimedia Appendix 1 Assignment of CHARISMHA function type groups versus subject areas (both assigned manually, one hit per dimension allowed). CHARISMHA: Chances and Risks of Mobile Health Apps.

Multimedia Appendix 2 Descriptive breakdown by manually assigned function type group (as defined in the CHARISMHA study). CHARISMHA: Chances and Risks of Mobile Health Apps.

Multimedia Appendix 3 Description of cardiology-related apps broken down by manually defined topics. Topics with 10 or less apps were not listed.

## Abbreviations

API: application interface
CE: Conformité Européenne
CHARISMHA: Chances and Risks of Mobile Health Apps
ECG: electrocardiography
FDA: Food and Drug Administration
IQR: interquartile range
NLP: natural language processing
SARASA: semiautomated retrospective App Store analysis
