# Supplementary material for: Description of Cardiological Apps From the German App Store: Semiautomated Retrospective App Store Analysis
Source: JMIR Mhealth Uhealth. 2018 Nov 20;6(11):e11753. doi: 10.2196/11753 (PMC6280035; doi:10.2196/11753)
Supplement: Multimedia Appendix 1 [file mhealth_v6i11e11753_app1.pdf]

Multimedia Appendix 1. Assignment of CHARISMHA [1] function type groups vs. subject areas (both assigned manually, one hit per dimension allowed).

	Function type groups as defined in CHARISMHA (manually assigned)						Cumulated number of apps per topic (out of N=335)	
	Provision of information	Support	Data acquisition, processing and evaluation	Calendar and appointment related	Other	Administrative use	Number of apps	Percentage of apps
**Topic (manually assigned)**								
Health data	3	22	28	10	—	4	67	20
Other	31	3	8	—	2	1	45	13.4
Emergencies	32	7	—	—	5	—	44	13.1
Blood pressure	1	4	5	26	—	—	36	10.7
Complementary medicine	7	24	—	—	—	—	31	9.3
Metabolism	4	2	3	9	—	—	18	5.4
ECG	7	—	6	1	1	—	15	4.5
Conferences	14	—	—	—	—	—	14	4.2
Medication	5	2	2	3	—	—	12	3.6
Atlases	10	—	—	—	—	—	10	3
Communication	2	—	1	1	6	—	10	3
Medical practice or hospital	7	—	1	—	2	—	10	3
Psyche	—	4	2	—	—	—	6	1.8
Sleep	4	—	2	—	—	—	6	1.8
Animals	1	1	1	1	—	—	4	1.2
Neurology	2	—	1	—	—	—	3	0.9
Women	—	2	—	—	—	—	2	0.6
Nutrition	—	—	—	1	—	—	1	0.3
Fitness	—	1	—	—	—	—	1	0.3
**Cumulated number of apps per function type (out of N=335)**								
Cumulated number of apps	130	72	60	52	16	5	335	100
Percentage of apps per function type	38.8	21.5	17.9	15.5	4.8	1.5	100	

1. Albrecht U-V, Höhn M, von Jan U. Kapitel 2. Gesundheits-Apps und Markt. In: Albrecht U-V, editor. Chancen und Risiken von Gesundheits-Apps (CHARISMHA), engl Chances and Risks of Mobile Health Apps (CHARISMHA). Hannover: Medizinische Hochschule Hannover; 2016. 62-82
