# Supplementary material for: Description of Cardiological Apps From the German App Store: Semiautomated Retrospective App Store Analysis
Source: JMIR Mhealth Uhealth. 2018 Nov 20;6(11):e11753. doi: 10.2196/11753 (PMC6280035; doi:10.2196/11753)
Supplement: Multimedia Appendix 2 [file mhealth_v6i11e11753_app2.pdf]

Multimedia Appendix 2. Descriptive breakdown by manually assigned function type group (as defined in the CHARISMHA study [1]).

App demographics	Cardiology related apps (N=335)	Provision of information (N = 130)	Data acquisition, processing and evaluation (N = 60)	Administrative use (N = 5)	Calendar and appointment related (N = 52)	Support (N = 72)	Other (N = 16)
Overall age of the apps in months, median (IQR[a])	39.25 (48.39)	33.38 (52.36)	44.59 (41.07)	34.42 (36.10)	53.38 (36.63)	41.92 (46.30)	14.67 (26.64)
Age in months (current version only), median (IQR)	7.73 (20.20)	9.47 (20.30)	7.66 (20.28)	3.08 (0.78)	6.05 (26.13)	7.40 (12.42)	5.83 (8.10)
File size in megabytes, median (IQR)	30.25 (53.58)	28.79 (54.58)	16.66 (36.27)	9.28 (53.79)	15.64 (44.31)	52.59 (51.28)	28.86 (84.84)
**Price in Euros (€)**							
Number of paid apps and percentage of total, n (%)	91 (27.2)	33 (25.4)	19 (32)	–	18 (35)	21 (29)	–
Price, median (IQR)	3.49 (4.70)	8.99 (18.70)	3.49 (3.35)	–	2.89 (2.10)	3.49 (3.20)	–
Price range (€)	0.49-249.99	0.49-249.99	1.09-229.99	–	0.99-6.99	1.09-10.99	–
Length of the store description (number of characters), median (IQR)	1630 (1585.50)	1254.00 (1212.75)	1644.00 (1409.75)	1957 (2133.00)	1860.50 (1345.25)	2231.50 (1424.50)	1510.00 (1805.50)
**Star ratings (current version)**							
Rated apps, n (%)	144 (43.0)	44 (33.8)	30 (50)	–	28 (54)	35 (49)	7 (44)
Median rating (IQR)	4.50 (2.00)	4.50 (1.00)	4.50 (1.50)	–	4.00 (1.50)	3.50 (2.00)	3.50 (1.75)
Maximum number of ratings (n)	645	592	390	–	140	645	5
Number of ratings, median (IQR)	3.00 (8.00)	44; 2.00 (4.00)	6.00 (38.00)	–	5.00 (8.25)	4.00 (10.00)	2.00 (2.00)
**Overall star ratings (all versions)**							
Rated apps, n (%)	173 (51.6)	54 (41.5)	32 (53)	–	32 (62)	56 (78)	9 (56)
Median rating (IQR)	4.00 (1.50)	4.00 (1.50)	4.25 (1.50)	–	4.00 (1.12)	3.50 (1.00)	4.00 (2.00)
Maximum number of ratings (n)	6881	1233	5079	–	1018	6881	25
Number of ratings, median (IQR)	14.00 (49.00)	6.50 (16.50)	19.50 (572.75)	–	21.00 (220.25)	19.50 (56.75)	5.00 (4.00)

[a]IQR: interquartile range.

1. Albrecht U-V, Höhn M, von Jan U. Kapitel 2. Gesundheits-Apps und Markt. In: Albrecht U-V, editor. Chancen und Risiken von Gesundheits-Apps (CHARISMHA), engl Chances and Risks of Mobile Health Apps (CHARISMHA). Hannover: Medizinische Hochschule Hannover; 2016. 62-82
