# Supplementary material for: Description of Cardiological Apps From the German App Store: Semiautomated Retrospective App Store Analysis
Source: JMIR Mhealth Uhealth. 2018 Nov 20;6(11):e11753. doi: 10.2196/11753 (PMC6280035; doi:10.2196/11753)
Supplement: Multimedia Appendix 3 [file mhealth_v6i11e11753_app3.pdf]

Multimedia Appendix 3. Description of cardiology-related apps broken down by manually defined topics. Topics with 10 or less apps were not listed.

App demographics	Cardiology-related apps (N=335)	Health data (N = 67)	Other (N = 45)	Emergencies (N = 44)	Blood pressure (N = 36)	Complementary medicine (N = 31)	Metabolism (N = 18)	ECG (N = 15)	Conferences (N = 14)	Medication (N = 12)
Overall age of the apps in months, median (IQR[a])	39.25 (48.39)	34.42 (38.58)	41.29 (46.19)	31.69 (60.94)	52.68 (37.49)	58.49 (47.48)	52.11 (48.71)	50.74 (31.85)	22.14 (26.33)	33.51 (36.51)
Age in months (current version only), median (IQR)	7.73 (20.20)	5.00 (18.08)	11.36 (25.36)	6.44 (10.58)	5.94 (27.88)	9.60 (9.83)	7.70 (25.08)	9.40 (19.89)	18.79 (24.91)	3.83 (12.16)
File size in megabytes, median (IQR)	30.25 (53.58)	36.34 (59.13)	30.60 (73.62)	16.76 (27.66)	9.65 (38.09)	68.28 (38.98)	24.55 (42.42)	22.57 (60.07)	51.43 (22.65)	27.73 (65.77)
**Price in Euros (€)**										
Number of paid apps and percentage of total, n (%)	91 (27.2)	15 (22)	9 (20)	7 (16)	14 (39)	16 (52)	5 (28)	7 (47)	–	1 (8)
Price, median (IQR)	3.49 (4.70)	3.49 (3.20)	6.99 (7.00)	6.99 (7.30)	2.29 (2.40)	4.49 (3.20)	3.99 (2.20)	20.99 (119.85)	–	1.09 (0.00)
Price range (€)	0.49-249.99	1.09-16.99	1.09-19.99	1.09-32.99	0.99-6.99	2.29-28.99	1.09-6.99	1.09-249.99	–	1.09
Length of the store description (number of characters), median (IQR)	1630 (1585.50)	1681 (1783.50)	1259 (1058.00)	1295.00 (1393.25)	1941.00 (1199.00)	2600 (736.50)	2003.50 (1268.00)	1065 (347.50)	749.00 (95.25)	2023.00 (1829.50)
**Star ratings (current version)**										
Rated apps, n (%)	144 (43)	31 (46)	13 (29)	14 (32)	25 (69)	13 (42)	12 (67)	3 (20)	3 (21)	6 (50)
Median rating (IQR)	4.50 (2.00)	3.50 (1.50)	4.00 (2.00)	4.50 (0.88)	4.50 (1.50)	5.00 (3.00)	4.00 (0.75)	5.00 (0.50)	5.00 (0.25)	4.00 (1.38)
Maximum number of ratings (n)	645	645	20	158	150	35	35	33	3	27
Number of ratings, median (IQR)	3.00 (8.00)	5.00 (50.00)	1.00 (1.00)	4.00 (5.50)	5.00 (7.00)	2.00 (4.00)	2.00 (4.00)	6.00 (9.50)	2.00 (1.00)	10.50 (4.75)

App demographics	Cardiology-related apps (N=335)	Health data (N = 67)	Other (N = 45)	Emergencies (N = 44)	Blood pressure (N = 36)	Complementary medicine (N = 31)	Metabolism (N = 18)	ECG (N = 15)	Conferences (N = 14)	Medication (N = 12)
**Overall star ratings (all versions)**										
Rated apps, n (%)	173 (51.6)	34 (51)	18 (40)	19 (43)	27 (75)	20 (65)	14 (78)	3 (20)	3 (21)	6 (50)
Median rating (IQR)	4.00 (1.50)	3.50 (1.50)	3.75 (1.88)	4.00 (1.25)	4.00 (1.00)	4.00 (0.62)	4.00 (0.88)	5.00 (0.25)	5.00 (1.00)	4.25 (0.88)
Maximum number of ratings (n)	6881	6881	59	1233	795	265	468	14	7	996
Number of ratings, median (IQR)	14.00 (49.00)	48.00 (623.25)	5.50 (13.00)	14.00 (14.50)	18.00 (54.00)	14.50 (28.00)	23.00 (114.75)	12.00 (4.00)	2.00 (3.00)	85.50 (677.50)

[a]IQR: interquartile range.
